# Supplementary figures and images for: Computational modelling of muscle fibre operating ranges in the hindlimb of a small ground bird (Eudromia elegans), with implications for modelling locomotion in extinct species
Source: PLoS Comput Biol. 2021 Apr 1;17(4):e1008843. doi: 10.1371/journal.pcbi.1008843 (PMC8016346; doi:10.1371/journal.pcbi.1008843)

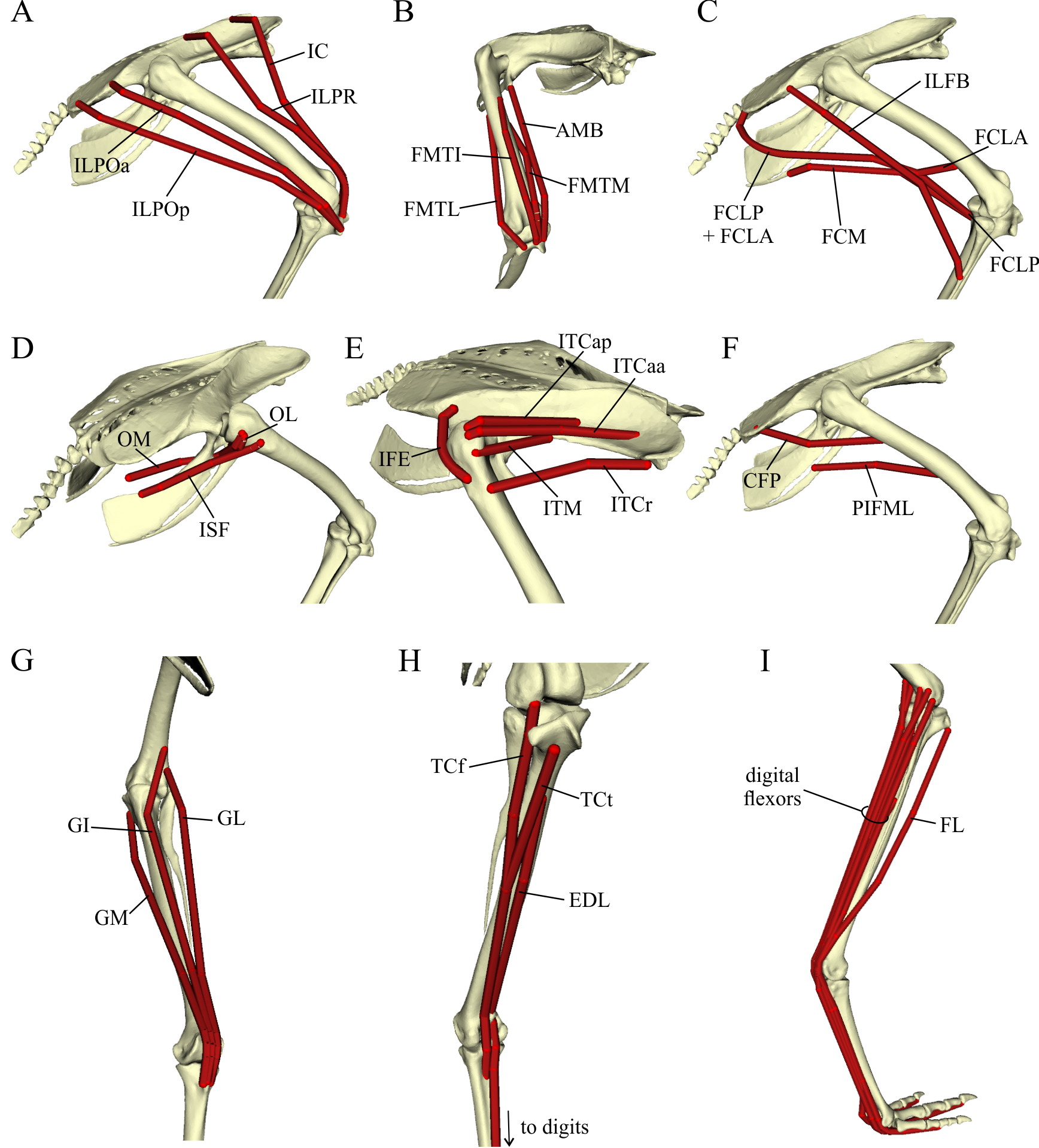

Supplement: S1 Fig — (A) Iliotibiales. (B) Deep knee extensors. (C) ‘Hamstrings’. (D) Deep external rotators. (E) Deep internal rotators. (F) Caudal hip extensors. (G) Gastrocnemii. (H). Ankle flexors. (I) Ankle extensors and metatarsophalangeal plantarflexors; the digital flexors are the FDL, FHL, FP2, FPP2, FP3, FPP3 and FP4. (TIF) [file pcbi.1008843.s001.tif]

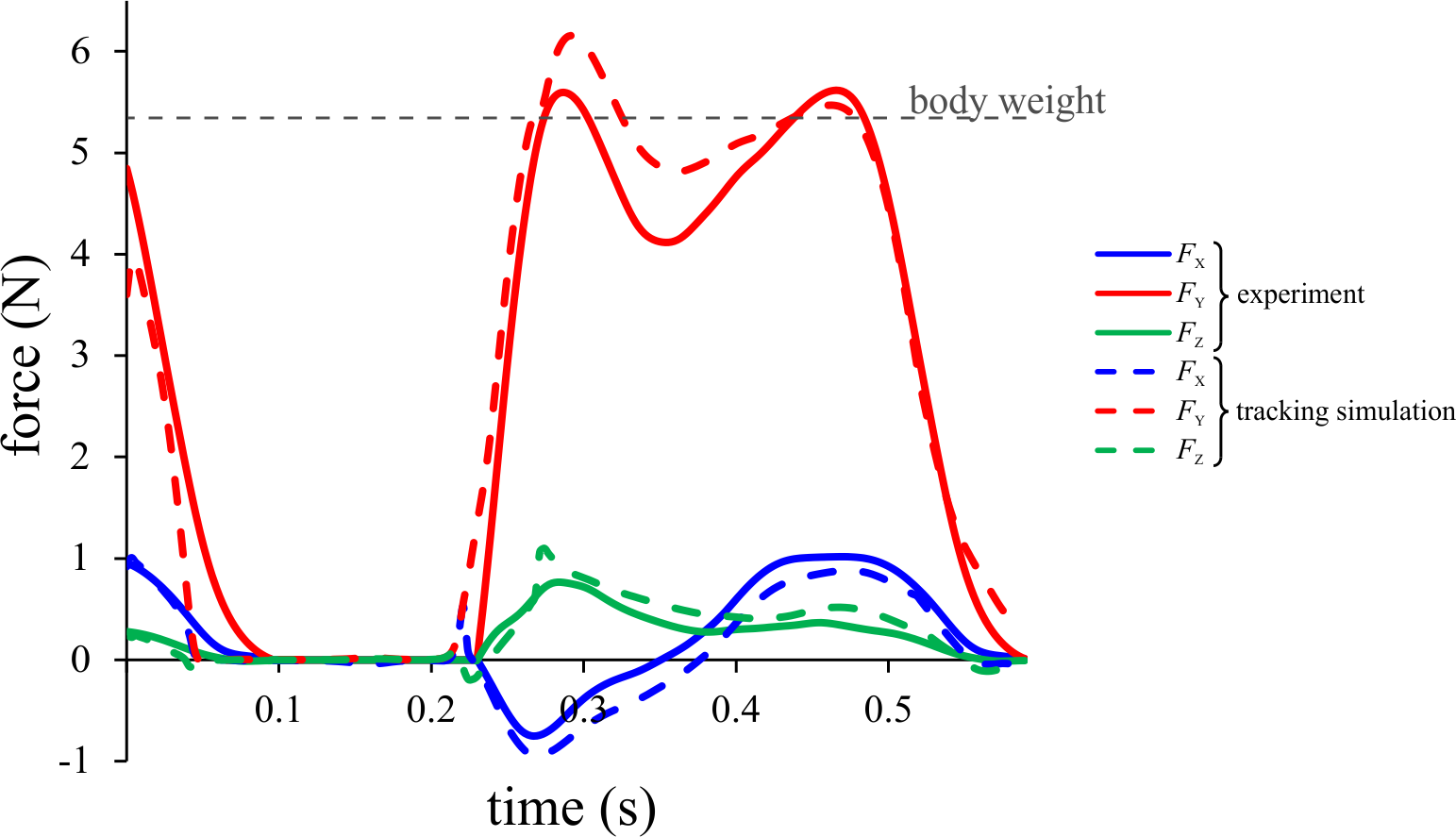

Supplement: S2 Fig — The running trial is not shown since no experimental data was able to be collected here. (TIF) [file pcbi.1008843.s002.tif]
