# Supplementary material for: Computational modelling of muscle fibre operating ranges in the hindlimb of a small ground bird (Eudromia elegans), with implications for modelling locomotion in extinct species
Source: PLoS Comput Biol. 2021 Apr 1;17(4):e1008843. doi: 10.1371/journal.pcbi.1008843 (PMC8016346; doi:10.1371/journal.pcbi.1008843)
Supplement: Attachment — Submitted filename: Review1.pdf [file pcbi.1008843.s008.pdf]

Review:

This paper describes the generation of a musculoskeletal model of the tinamou, a ground bird. The musculoskeletal model was created using imaging, and cadaver studies. Walking and running data was also created using XROMM based motion capture and force plates on a walkway and a treadmill (without force plates). The goal was to investigate length changes of the muscle fibre length during walking and running, as well as during the full range of motion using static optimizations. It was found that the fibre length remains within 50% to 120% of the optimal fibre length, which is a somewhat smaller range than expected. Furthermore, the operating range was more on the ascending limb, below optimal fibre length of the force-length curve, which has implications for estimating optimal fibre length in extinct animals.

This is very interesting work, and the paper is well written and I enjoyed reading it. However, I still have a couple of questions/concerns regarding some of the modelling/simulation decisions and presentation of results.

Major comments:

- The cut-off frequency when filtering the kinematic and kinetic data is different. According to [1], inconsistencies can arise when the cut-off frequency for kinematic and kinetic data is not the same. Please elaborate on the choice of cut—off frequency.
  [1] Derrick, T. R., van den Bogert, A. J., Cereatti, A., Dumas, R., Fantozzi, S., & Leardini, A. (2020). ISB recommendations on the reporting of intersegmental forces and moments during human motion analysis. Journal of Biomechanics, 99, 109533.
- The methods section is very long. The methods are explained very well which makes the study is reproducible. However, the problem is that I forgot what was written in the introduction/early methods by the time I started reading the results. Therefore, the paper would be more readable if some details are moved to a supplement. E.g. the specifics of recording necessary only for reproduction, or the motivation for using simulations instead of inverse dynamics. Relatedly, I was surprised that the figures showing the results for walking were added as supplement, since I would consider those more important than some of the details mentioned in the methods section.
- Line 585 & 743: It seems that the objective weights were hand-tuned, both for the simulations to generate moments and muscle lengths. I think it is important for non-experts of these optimizations to understand that these choices might affect the results. Please mention this, e.g. in the discussion.
- Page 25: Why was an estimated GRF vector created and tracked during the running trial? Did this yield better results than tracking only the kinematic data available from the study?
- Page 30, item 4: since the aim of the paper is to explore if the muscle length during gait is on the descending limb of the force-length curve, it seems unfair to actually penalize this behaviour. Did you test to see if a smaller weight increased the normal operating range of the muscle fibre length?
- Relatedly, line 861: again results with high fibre lengths were disregarded, which seems again unfair with respect to the aim of investigating the behaviour on the force-length curve.
- Why was it chosen to use the model with 3 DOF ankle and knee instead of the one using only flexion/extension? One degree of freedom in the ankle and knee is common in human modelling too and would remove the need for the residual actuation. The discussion also mentions that similar results were obtained with the model with less DOF. It is usually advised to use the simplest model to generate the necessary results, which I would think is the model without these four extra degrees of freedom.

- Figure 4 and 6: the titles of the plots were hard to read. This might be due to how they are processed by the PLOS software.
- Figure 4-5: What were the initial/boundary conditions to the inverse simulation problem to find the muscle forces? The results do not seem periodic.
- Figure 6: Please elaborate on the small range of the ILPOa and FMTL. The FMTL was mentioned in the figure caption but I did not understand.
- Lines 880-902/Figure 7 and 8. I did not understand the significance of these results with respect to the aims mentioned in the introduction, because those were already met using the other methods/results. Perhaps consider expanding the aims.

Minor comments:

- Introduction: the references are generally put at the end of the sentence, but cover different options. For example, in lines 136-139, it would be nice, if possible, to separate the references to sensitivity analysis on muscle-tendon function [xx], patterns of muscle recruitment [yy], and whole organism performance [zz]. I also noticed this in lines 93-94, 114-116, 148-150.
- Related to the long methods section: by page 28, when the optimization of the MTU lengths is discussed, I was not sure anymore how each parameter was determined. Perhaps a table with a list of parameters and how they are determined would help.
- Figures general: I found it hard to find the different muscles in the figures. I am not an expert on bird anatomy, and I guess they are ordered in some way that makes sense anatomically. It might be helpful to have pointers (e.g. top right, or maybe similar to a chess board or map with letters and numbers) to be able to easily find the muscles.
- Line 728: why was the data filtered again before the inverse simulation?
- Line 1230-1242: it was chosen to optimize the running and walking trial simultaneously. In machine learning, where data is used to derive model parameters, data is often separated in a testing and training dataset, such that the model is optimized with the training data and validated on the testing data, which is “new”. Have you also considered this approach?
