# Supplementary material for: Computational modelling of muscle fibre operating ranges in the hindlimb of a small ground bird (Eudromia elegans), with implications for modelling locomotion in extinct species
Source: PLoS Comput Biol. 2021 Apr 1;17(4):e1008843. doi: 10.1371/journal.pcbi.1008843 (PMC8016346; doi:10.1371/journal.pcbi.1008843)
Supplement: Attachment — Submitted filename: Review2.pdf [file pcbi.1008843.s012.pdf]

Thank you to the reviewers for the responses. I feel that the changes have improved the paper's readability. As said before, the topic is very interesting. By the way, I was not able to see the yellow highlights in the manuscript.

Major comment:

"C1: The cut-off frequency when filtering the kinematic and kinetic data is different. According to [1], inconsistencies can arise when the cut-off frequency for kinematic and kinetic data is not the same. Please elaborate on the choice of cut—off frequency.

A1: Our kinematic (XROMM) and kinetic (forceplate) data were obtained via very different means, rather than straight recording via a semi-automated motion capture protocol as is the norm in human studies. This consequently meant that the data had varying levels of 'quality' and noise. For example, the forceplates we used were quite old and prone to drift and noise, and so we erred on the side of caution and used a more aggressive filtering (lower cutoff frequency) for those data (clarifying statement now included on p. 15, line 393-397). We acknowledge that in a more ideal situation – and certainly one where we would be collecting data for many trials in a comparative analysis – a more consistent cutoff frequency would be preferred. Nonetheless, the cutoff frequencies that we ultimately used are similar to those used in previous studies of bird locomotion (e.g., Bishop et al., 2018), and ultimately our resulting kinematic and kinetic profiles are highly comparable to previously published data, so we have little concern with this aspect of the study."

I think I mostly agree with the author's response. However, at the same time I am not completely sure that we understand each other completely. Therefore, I have elaborated a bit more on this below:

"rather than straight recording via a semi-automated motion capture protocol as is the norm in human studies" From this, I thought that the kinetics and kinematics were recorded in a separate trial. However, from the manuscript I understand that the kinematics and kinetics were measured simultaneously for the same stride. So I do not understand this part of the comment. Were the different recordings not synchronized? I think that that is the only difference between this approach and commonly used human motion capture that would affect the filtering issue.

Furthermore, the issue with having a different cut-off frequency for motions and forces, is that in this case you will filter out force peaks that happen at e.g. 18 Hz (the cut-off frequency is 15 Hz), while the corresponding 18 Hz motion is still there (because the cut-off frequency is 20 Hz). This will cause inconsistencies in the inverse dynamics results, because the motions that go into the multibody equation might not correspond to forces that go into the same equation. Furthermore, when data is not synchronized or a different time delay is introduced due to different cut-off frequencies, this would be even worse, because then the timing of motions is off. For example, in human gait, foot flat after heel strike would have to be done actively if the corresponding ground reaction force is not present, which could greatly affect joint moments. I suspect that this filtering inconsistency might also partly cause the high results in the residual reduction algorithm. However, I would say that this approach is fine for the optimal control problem, because there the experimental results are not directly inserted into the equation but tracked as best as possible.

Minor comments:

A couple of times, I was a bit confused because so many processing steps are required, and I got lost a bit about exactly what was used where.

- Residual reduction algorithm: why change the mass when this is actually measured exactly? Or was this only measured for the bird used for modelling and not for kinematics? Also considering the comment above, I am wondering a bit if you are correcting a measurement

error by adapting the model, so that the residual reduction algorithm cancels out one error by introducing another error.

- Did the optimal control use the model that was changed after residual reduction?
- Line 676-678: How did the optimal control produce a musculoskeletal model with altered mass properties?
- Equation 4, line 796-802. You chose a high weight to ensure that reserve actuators were used as little as possible. Did you check if reserve actuators were used when activation was not yet maximal?
- Line 1000 – 1006: based on the reviewer comments that I had already read, I felt that here it would make sense to already add that these moments are caused by passive tissues, but then I noticed that this was addressed later on in the manuscript. However, since the manuscript is rather long, it might be helpful for future readers to connect these two paragraphs a bit more.
- Line 1072- 1076: again, here I was wondering if there was an explanation, which I later found in the discussion. Again, it might be helpful to connect the paragraphs in the results and discussion.
- Typo line 1188 (waking)
- Section "Static simulations", line 1170: I got lost a little bit here with all the different "l"s. I think readability could be improved when full names "e.g. optimal fibre length" are added to the variable names.
- 1376 – 1379: would you conclude after this study that a one DOF ankle and knee is appropriate for musculoskeletal models of birds?
