# Supplementary material for: Computational modelling of muscle fibre operating ranges in the hindlimb of a small ground bird (Eudromia elegans), with implications for modelling locomotion in extinct species
Source: PLoS Comput Biol. 2021 Apr 1;17(4):e1008843. doi: 10.1371/journal.pcbi.1008843 (PMC8016346; doi:10.1371/journal.pcbi.1008843)
Supplement: Attachment — Submitted filename: review_Bishop_etal_PlosCompBio2020.docx [file pcbi.1008843.s009.docx]

Reviewer evaluation

In this well written, thoroughly conducted study, the authors explore the range of fibre lengths visited in tinamou during walking and running, and address the methodological question of whether and how it is possible to estimate optimal muscle fibre length from joint range of motion. In addition, they expand state-of-the-art methodology in musculoskeletal modeling. The authors went to great lengths in their analysis to ensure consistency within their model. The latter was, in part, necessary because of the limited amount of experimental data (essentially N=1, in terms of the kinetics and kinematics) that was collected for this study. This is unfortunate because it leaves open the possibility that the same study conducted on data from a different individual would yield different results, i.e. the results presented here may not be robust against repetition. Next to concerns regarding the reproducibility of the results, there are several other questions/concerns which should be addressed before publication, please see ahead. Notwithstanding these concerns, this study makes a substantial contribution to muscolosketal modeling in general, and of tinamou in particular, which opens up the possibility for future (modeling) research on this bird, and on other (extinct) species.

Reviewer summary

In this study, the authors aimed to develop and validate a musculoskeletal model of a small bird (tinamou), which they subsequently used to explore the relationship between limb range of motion and muscle fibre length range.

They performed morphological measurements on euthanized tinamou and performed kinetic and kinematic measurements on walking and running tinamou. The morphological measurements were used to formulate and parameterize a 26 DOF skeletal model, feed this model with muscle pathing, and provide initial guesses for muscle’s structural parameters. The kinetic and kinematic measures were combined in a way that produced model based joint angles and joint moments as a function of time that were dynamically consistent with the ground reaction forces. Subsequently, an optimal control problem was solved for both the running and the walking trial simultaneously, to find muscle states as a function of time that satisfied the kinematics and kinetics of the model, and obeyed Hill-type muscle dynamics. In this optimization, optimal muscle fibre length and tendon slack length were treated as free parameters and could vary from their anatomically based initial values. The optimization results were found to be in reasonable agreement with (scarce) experimental data from the literature (from a different type of bird) in terms of muscle fibre length (i.e. model “contractile element” length) over time (quantified by sonomicrometry) and muscle activation over time (quantified by EMG). The normalized fibre lengths visited during these trials maximally ranged from 0.5-1.2, with some muscles using a greater portion of this range than others.

Next, the authors randomly chose 5000 static poses for a reduced (6 DOF) version of the lower limb model, spanning the full range of motion of this 6 DOF model. For each of these poses they solved various versions of the muscle load sharing problem to establish the maximal range of fibre lengths that could conceivably be visited. The range of fibre lengths visited in all poses was compared to the common presumption expressed in the literature that normalized muscle fibre length ranges from 0.5 to 1.5 over the full range of motion of a joint. It was found that this presumption does not hold for many muscle’s in their model. Lastly, the authors evaluated to what extent changes in joint angles are lin2early related to changes in muscle fibre length, i.e. to what extent non-constant moment arm, tendon and pennation angle influence this relationship. From their static simulation results, they computed the least squares optimal, linear transformation between muscle fibre length and joint angle, and report the differences between the linear model and the simulation results for each muscle. The linearity of this relation is left to the reader to evaluate. Their results may have implications for the estimation of optimal muscle fibre length in extinct/extant species.

Major Comments

It seems that the authors very carefully conducted each analysis step. However, there were very many steps. It is not clear to me to what extent arbitrary choices throughout the analysis may have influenced the results, especially considering the limited amount of experimental data that was available. Can you make a case for why the results of this study are nonetheless reproducible? For example: Missing sections of kinematics sequences; L335-350. The description of three levels of hierarchy in filling in missing data suggest that all three levels were used/needed. Was it evaluated to what extent the decisions made during this process influenced the results? E.g. taking different stride portions to fill in these data? In addition, such an analysis would give some indication of the robustness of the results.

Reserve actuators 1) I agree with your interpretation that the reserve actuators may represent moments generated by ligaments or joint capsula that were not explicitly modelled (L1290-1300). If these indeed represent passive structures, and given that we are considering near periodic motion (I think, but see minor point ahead), the net work done by these reserve actuators during one complete stride should at best be zero or if anything slightly negative. Can you check in your results if the work done by these reserve actuators during a complete stride is indeed close to zero/negative, ie the reserve actuators represent some (unmodelled) conservative / dissipative force?

Reserve actuators 2) L945-950. Apparently producing the required joint moments using muscles alone, even for DOF’s that were well-spanned by the muscles, resulted in unrealistic results. This might be viewed as an important indication that something is missing in the model, e.g. maximum forces may have been too low, or something else might be missing. What are your thoughts on this?

Fmax estimation. L1230-1240, in relation to figure 4. In figure 4, the activation seems to ‘clip’ or nearly clip for some muscles during the stance phase, which might be the reason why reserve actuators have to be invoked. Apart from the latter consequence, this also suggests that the running trial represented an activity that is close to the maximum effort capability of the animal. Considering the animal was moving in more or less steady state at moderate speed, this seems highly unlikely, and suggests that for instance your maximum force values might be underestimated, or some other (dynamical) muscle properties are misestimated. Do you agree with this? If so, were in the model do you suspect modifications are required? (Note that your maximal force values depend on L0. The simulation based estimated values for L0 were higher than the anatomical based L0, which were used to compute the estimates for maximum force. I believe this means basing maximum forces on simulation based L0 values would if anything decrease the Fmax values.)

Minor Comments/questions/suggestions:

To facilitate comparison with other literature/species, consider (also) reporting non-dimensionalized values throughout the manuscript (e.g. non-dimensionalized running/walking velocity, ground reaction force, etc.)

Do you have any indication to what extent the surgery (6 days recovery time) influenced the walking pattern of the tinamou, e.g. comparison of video data before/after surgery?

Fig2. For both L* and Lt the scaling values are >1. Is this an indication that the muscle’s origin-insertion lengths in the model (for a similar anatomical pose) were on average larger than the origin insertion lengths during the anatomical measurements? In other words, could this result have been different if the muscles were a bit ‘stretched’ during the anatomical measurements?

L537; Vmax and Fmax were the same for all muscles, but there may exist some between-muscle variance in dynamic response, for instance due to differences in fibre type distributions. Can you make a case against the alternative hypothesis that the differences in (optimized) morphological properties between muscles do not reflect real morphological differences but are due unmodelled differences in the dynamical properties of the muscles?

L1124. For L*<0.5 the force producing capacity is ~0, and thus the force, and in particular tendon length (and thus L*) is dependent on the (arbitrary) numerical implementation of 0 muscle fibre force. Considering this, are the results for these 6 muscles meaningful? If not, consider removing them.

L564: total body mass increased by 2.1 % to resolve dynamic inconsistency; this is surprising as I expect both your scale (on the basis of which animal mass (table 1) was determined) and your force plate to have an accuracy that is better than this 2.1 % BW. Could you explain?

Table 2. I suspect that for the given methodology the number of significant digits provided for the variables in this table may not represent the accuracy / degree of certainty by which these values were measured / estimated (e.g. anatomically measured pennation angle accuracy of 0.01 deg, anatomically measured fibre length accuracy of 0.01 mm, or Fmax estimation accuracy of 1 mN…) . If this were to be the case, please correct the number of significant digits accordingly.

L611; 1) Here the modifications to GRM are not reported, which you may argue represent some dynamic inconsistency, or at least a modification to COP trajectories. Please also report the magnitude of these modifications. 2) “The greatest modification …” is not precise, as that refers to the greatest modification in terms of the RSME. Please correct so the reader understands that the greatest modification at any time node could have been larger than 10 % BW, or report this greatest modification (as the RMSE values are already reported in the table).

Please provide a figure showing the (non)modified GRF’s as function of gait cycle. This will help the reader get a better appreciation of the data and facilitates comparison to (future) literature.

L746. You mention the optimal control problem was subject to state continuity across trials. What does this mean? I initially thought this means the state variables were periodic, but looking at Figs 4 and 5 this does not appear to be case. Also, I am not sure whether the kinematics/kinetics of the skeletal part of the model are (enforced to be) periodic (as suggested by the kinematics cartoons)? Please clarify.

L754-780. I appreciate the elaborate justification of the weight factors used. To facilitate comparison between them consider rescaling the weight factors in this section to similar dimensionless units, e.g. in comparing activation (term 1, ranging for 0-1) to reserve actuators (term 2) it might help to scale the weight on the reserve actuator force/activation to maximal reserve actuator force, or maximal joint moment or similar.

L800-820; please state explicitly that this results in a 6 DOF joint space from which the 5000 random positions were drawn.

Fig 6. Please indicate how these distributions are normalized; there is no scale on the y-axis.

L1140-1160. Differences between delta Lmt and delta Lm can be due to either pennation angle or tendon elongation. Please also mention the latter. Which of these two has the biggest contribution to the difference between delta Lmt and delta Lm? (perhaps this question is implicitly addressed in fig 7B., if so please make this clear)

Fig 7A 1) In the current figure it is not clear for which muscle’s there is a large discrepancy between delta_Lmt and delta_Lm. Consider plotting (in light of the question to what extent delta_Lm can be inferred from joint range of motion) the difference between delta_Lmt and delta_Lm, normalized by L0, for each muscle. Alternatively, consider adding labels to the points most saliently away from parity. 2) I would not expect the solutions for minimizing (sim1) and maximizing (sim2) activation to be similar (unless the resulting activations were similar), because higher activation implies higher force which results in more tendon elongation; I would thus expect the results for sim2 to be further away (more down) from parity. Similar argument goes for sim3 and sim4. Consider showing all results, or the most extreme results here.

Fig 7B. I assume the grey line shows the linear fit. Consider plotting instead the theoretical relationship with pennation angle, for an non-compliant tendon, in which case the scatter would be due to variations in tendon length / force. L1156. The reference to differences with the line of parity in A is confusing here as these differences arise both from tendon compliance and pennation angle. If the point of Fig 7B is that higher pennation angles leads to greater decoupling between Lmt and Lm I would suggest making this statement in words rather than in a figure, as this observation follows directly from the formulation of the muscle model.

Table 4. Please add means (sd) across the columns of this table; this may help the reader get a general sense of the data quickly.

L1213-1228. I appreciate the consideration for the future abilities of this new tuning approach. Please consider adding a cautionary note for the uninitiated reader, something like: ‘this new tuning also affords the possibility of patching errors made elsewhere in the procedure, and unintentionally reaching spurious conclusions’, or something along similar lines.

L1364. “ … further paints a picture of structural conservatism across evolutionary timescales”. This statement is not strictly supported by data from the current study, please provide appropriate references.

Fig1. (legend) Please indicate which panel the scale bar serves.

Language:

Please re-check the paper thoroughly for English. At various points, the text could be more concise. For example:

L99; “muscles … coordinate” many would argue it is the brain or central nervous system which does the coordinating, consider reformulating.

L116; “such research”, it is not clear (at this point what) “such” is referring to, consider: ‘such experimental research’

L122; perspectiveS

L127; “muscles have been able to be” convoluted language, consider reformulating, eg ‘muscles have been classified according to … ‘

L135; remove “constituent”

L173. “In particular, this would test”. Unfortunate formulation, consider rewording, eg ‘These results demonstrate / using these results, we can test whether …’

L212; “recovered” I guess it didn’t exist before, consider rewording, eg ‘produced’?

L602-603; correct sentence ending in “zero residuals”.

L590; spell out GRF/GRM at first use (which may have been before this one).

L653; “external joint moments”. Imo the use of the word external could be confusing here, because external moments might be thought to refer to some moment acting from the environment onto a system, whereas joint moments per definition are moments acting from one segment onto another segment within the same skeletal system. Also, the contrast with internal might not be so clear. Consider rewording, eg muscle generated moments (if you want contrast with reserve actuators).

L787. Remove “then”.

L798. Convoluted language, consider reformulating, eg “the greatest range of physiologically possible muscle lengths …”

L809. “control”, consider: ‘actuate’.

L989. “other” 🡪 otherS

L1012. Modellling 🡪 modelling

L1031. add ‘are’.

L1175. Consider: ‘fitting was done on the results from simulations 1 and 2 together, and on …’

L1349. “… have been able to be …” consider: ‘were’
