# Supplementary material for: Computational modelling of muscle fibre operating ranges in the hindlimb of a small ground bird (Eudromia elegans), with implications for modelling locomotion in extinct species
Source: PLoS Comput Biol. 2021 Apr 1;17(4):e1008843. doi: 10.1371/journal.pcbi.1008843 (PMC8016346; doi:10.1371/journal.pcbi.1008843)
Supplement: Attachment — Submitted filename: Bishop_Responses_to_Reviwer_Comments.doc [file pcbi.1008843.s010.doc]

**Responses to Reviewers’ Comments**

We thank the reviewers greatly for their constructive and helpful comments. The original comments are listed below, and our response to each of these is given immediately after in blue font. Sections of the text where changes have been made in response to these comments are highlighted in yellow. These also include minor wording changes to improve readability or clarity.

**Reviewer 1**

General comments:

This paper describes the generation of a musculoskeletal model of the tinamou, a ground bird. The musculoskeletal model was created using imaging, and cadaver studies. Walking and running data was also created using XROMM based motion capture and force plates on a walkway and a treadmill (without force plates). The goal was to investigate length changes of the muscle fibre length during walking and running, as well as during the full range of motion using static optimizations. It was found that the fibre length remains within 50% to 120% of the optimal fibre length, which is a somewhat smaller range than expected. Furthermore, the operating range was more on the ascending limb, below optimal fibre length of the force-length curve, which has implications for estimating optimal fibre length in extinct animals.

This is very interesting work, and the paper is well written and I enjoyed reading it. However, I still have a couple of questions/concerns regarding some of the modelling/simulation decisions and presentation of results.

Major comments:

The cut-off frequency when filtering the kinematic and kinetic data is different. According to [1], inconsistencies can arise when the cut-off frequency for kinematic and kinetic data is not the same. Please elaborate on the choice of cut—off frequency.

[1] Derrick, T. R., van den Bogert, A. J., Cereatti, A., Dumas, R., Fantozzi, S., & Leardini, A. (2020). ISB recommendations on the reporting of intersegmental forces and moments during human motion analysis. Journal of Biomechanics, 99, 109533.

Our kinematic (XROMM) and kinetic (forceplate) data were obtained via very different means, rather than straight recording via a semi-automated motion capture protocol as is the norm in human studies. This consequently meant that the data had varying levels of ‘quality’ and noise. For example, the forceplates we used were quite old and prone to drift and noise, and so we erred on the side of caution and used a more aggressive filtering (lower cutoff frequency) for those data (clarifying statement now included on p. 15, line 393-397). We acknowledge that in a more ideal situation – and certainly one where we would be collecting data for many trials in a comparative analysis – a more consistent cutoff frequency would be preferred. Nonetheless, the cutoff frequencies that we ultimately used are similar to those used in previous studies of bird locomotion (e.g., Bishop et al., 2018), and ultimately our resulting kinematic and kinetic profiles are highly comparable to previously published data, so we have little concern with this aspect of the study.

The methods section is very long. The methods are explained very well which makes the study is reproducible. However, the problem is that I forgot what was written in the introduction/early methods by the time I started reading the results. Therefore, the paper would be more readable if some details are moved to a supplement. E.g. the specifics of recording necessary only for reproduction, or the motivation for using simulations instead of inverse dynamics. Relatedly, I was surprised that the figures showing the results for walking were added as supplement, since I would consider those more important than some of the details mentioned in the methods section.

As *PLOS Computational Biology* is an online-only journal, we don’t see much sense in moving large amounts of content to a second online document, and prefer to keep all the details together in the one place. However, we do acknowledge the issue noted by the Reviewer, and so to help the reader better follow the sequence of steps used, we have now created a new figure (Fig. 1) that schematically outlines the key steps of our workflow. Additionally, we have moved the figure for walking into the main text (Fig. 5) and cited it as appropriate.

Line 585 & 743: It seems that the objective weights were hand-tuned, both for the simulations to generate moments and muscle lengths. I think it is important for non-experts of these optimizations to understand that these choices might affect the results. Please mention this, e.g. in the discussion.

True, the specific values of the weightings were somewhat arbitrarily chosen, but their relative magnitudes do reflect our assessment of the relative importance of differing terms as per our study’s objectives. We have now raised this point in the Methods section immediately following our definition of the weightings used (p. 32, line 831-836).

Page 25: Why was an estimated GRF vector created and tracked during the running trial? Did this yield better results than tracking only the kinematic data available from the study?

Absolutely! Our representation of ground-foot contact, with a single sphere and (essentially untested) contact parameters, plus the less-than-ideal quality of the kinematic data, meant that the resulting GRFs produced in a kinematic-tracking-only simulation would have been awful. Tracking a (theoretically expected) GRF profile therefore helps keep the simulation grounded in greater biorealism. This has now been pointed out (p. 26, line 667-671).

Page 30, item 4: since the aim of the paper is to explore if the muscle length during gait is on the descending limb of the force-length curve, it seems unfair to actually penalize this behaviour. Did you test to see if a smaller weight increased the normal operating range of the muscle fibre length?

We initially did not have this term in the objective function, but a few muscles would optimize to be operating exclusively at very long normalized fibre lengths (some over 1.5), which we considered unreasonable. Hence the inclusion of this term. Moreover, the weighting value we ultimately settled upon represents our (admittedly) arbitrary compromise between avoiding this unrealistic behaviour and not adversely affecting the results obtained for other muscles. We have now explained this point (p. 31-32, line 815-820).

Relatedly, line 861: again results with high fibre lengths were disregarded, which seems again unfair with respect to the aim of investigating the behaviour on the force-length curve.

This is not quite true. We discarded these poses because they could not actually solve at all (i.e., MTU equilibrium was not possible), not because certain muscles had high normalized fibre lengths *per se*. Although, as originally noted, we suspect that the reason that these poses could not solve was because of high passive forces due to long normalized fibre lengths (in at least one muscle). We have now clarified this point accordingly (p. 35, line 917-920).

Why was it chosen to use the model with 3 DOF ankle and knee instead of the one using only flexion/extension? One degree of freedom in the ankle and knee is common in human modelling too and would remove the need for the residual actuation. The discussion also mentions that similar results were obtained with the model with less DOF. It is usually advised to use the simplest model to generate the necessary results, which I would think is the model without these four extra degrees of freedom.

Previous studies have shown that bird knees and ankles are much more mobile about non flexion–extension axes than the joints in humans, and given that so little has been investigated regarding musculoskeletal control in birds, we thought it would be better to include these joints to see what we get, rather than *a priori* exclude them from the outset. Indeed, we consider our reliance on reserve actuators to be a genuine, biologically meaningful result in this regard: although birds have more mobile knees and ankles, it appears that the control of these additional degrees of freedom still depends to a considerable degree on passive forces rather than active muscular force.

Figure 4 and 6: the titles of the plots were hard to read. This might be due to how they are processed by the PLOS software.

Yes, we also noted the same issue in the proof PDF of the submission, that the images were a bit blurry. The originally submitted images were of a higher quality, and we hope that in their final form their legibility will not be reduced.

Figure 4-5: What were the initial/boundary conditions to the inverse simulation problem to find the muscle forces? The results do not seem periodic.

The results were not periodic because these were based on experimental data for a single trial (for walking or running). The inverse simulation results (which simply take these data as input and solve the muscle redundancy problem) will therefore also not be periodic. The initial guess fed to the muscle redundancy solver was an estimate of muscle activations derived from a static optimization solution applied to the experimental data, as noted in the text. Other than that, no boundary conditions or similar constraints were imposed on the start or end of the solution sequence. We have now made a statement to that effect in the relevant section of the Methods (p. 30-31, line 787-790).

Figure 6: Please elaborate on the small range of the ILPOa and FMTL. The FMTL was mentioned in the figure caption but I did not understand.

We have now provided greater clarification in the caption (p. 45, line 1198-1202). We suspect that the narrow range of operating lengths for both muscles may be due to these MTUs’ actuator paths running close to the hip and/or knee joints, and so have a pretty low moment arm in flexion–extension; thus they do not undergo much whole-MTU length change to begin with.

Lines 880-902/Figure 7 and 8. I did not understand the significance of these results with respect to the aims mentioned in the introduction, because those were already met using the other methods/results. Perhaps consider expanding the aims.

The primary point of our static analyses was that our *in vivo* experimental data and inverse simulations only captured a fraction of the total range of joint poses (and combinations thereof) that the limb is capable of using, so these static analyses helped to fill in the gaps and examine fibre length patterns across the whole range of possible limb configurations. This point has now been reiterated and clarified in both the Introduction (p. 8, line 192) and Methods (p. 9, line 218-219, p. 33, line 851-854) sections, and the new schematic figure also helps convey this too.

Minor comments:

Introduction: the references are generally put at the end of the sentence, but cover different options. For example, in lines 136-139, it would be nice, if possible, to separate the references to sensitivity analysis on muscle-tendon function [xx], patterns of muscle recruitment [yy], and whole organism performance [zz]. I also noticed this in lines 93-94, 114-116, 148-150.

We initially tended to put references at the end of a sentence to aid readability, but have now followed the Reviewer’s suggestion and dispersed them throughout a sentence where relevant.

Related to the long methods section: by page 28, when the optimization of the MTU lengths is discussed, I was not sure anymore how each parameter was determined. Perhaps a table with a list of parameters and how they are determined would help.

As above, we have now created a new, schematic figure that helps convey how different steps (and the data derived from them) feed into one another, hopefully addressing this concern.

Figures general: I found it hard to find the different muscles in the figures. I am not an expert on bird anatomy, and I guess they are ordered in some way that makes sense anatomically. It might be helpful to have pointers (e.g. top right, or maybe similar to a chess board or map with letters and numbers) to be able to easily find the muscles.

We have now created a new figure S1 that provides a key to all the muscles in the model. Regarding the presentation of data in the main text figures, muscles are generally ordered in a proximal-to-distal fashion.

Line 728: why was the data filtered again before the inverse simulation?

This was simply to remove additional noise/artifact that crept in (due to the long-winded, multi-step process we had to use) at different stages. Especially given that we only had one trial each for walking and running to work with, we did not feel confident that each little bump in our curves was necessarily meaningful, and thus we kept our analyses and simulations focused on the major patterns of change at hand. A statement to this effect has now been made (p. 29, line 757-761).

Line 1230-1242: it was chosen to optimize the running and walking trial simultaneously. In machine learning, where data is used to derive model parameters, data is often separated in a testing and training dataset, such that the model is optimized with the training data and validated on the testing data, which is “new”. Have you also considered this approach?

Ideally, we definitely would like to do this, as it would enhance the rigour of the approach used. We first need to have *n* > 1 for each gait though! Now that we have established a workflow of sorts for small birds, this large-scale type of investigation will hopefully be more achievable in future.

**Reviewer 2**

General comments:

In this well written, thoroughly conducted study, the authors explore the range of fibre lengths visited in tinamou during walking and running, and address the methodological question of whether and how it is possible to estimate optimal muscle fibre length from joint range of motion. In addition, they expand state-of-the-art methodology in musculoskeletal modeling. The authors went to great lengths in their analysis to ensure consistency within their model. The latter was, in part, necessary because of the limited amount of experimental data (essentially N=1, in terms of the kinetics and kinematics) that was collected for this study. This is unfortunate because it leaves open the possibility that the same study conducted on data from a different individual would yield different results, i.e. the results presented here may not be robust against repetition. Next to concerns regarding the reproducibility of the results, there are several other questions/concerns which should be addressed before publication, please see ahead. Notwithstanding these concerns, this study makes a substantial contribution to musculoskeletal modeling in general, and of tinamou in particular, which opens up the possibility for future (modeling) research on this bird, and on other (extinct) species.

In this study, the authors aimed to develop and validate a musculoskeletal model of a small bird (tinamou), which they subsequently used to explore the relationship between limb range of motion and muscle fibre length range.

They performed morphological measurements on euthanized tinamou and performed kinetic and kinematic measurements on walking and running tinamou. The morphological measurements were used to formulate and parameterize a 26 DOF skeletal model, feed this model with muscle pathing, and provide initial guesses for muscle’s structural parameters. The kinetic and kinematic measures were combined in a way that produced model based joint angles and joint moments as a function of time that were dynamically consistent with the ground reaction forces. Subsequently, an optimal control problem was solved for both the running and the walking trial simultaneously, to find muscle states as a function of time that satisfied the kinematics and kinetics of the model, and obeyed Hill-type muscle dynamics. In this optimization, optimal muscle fibre length and tendon slack length were treated as free parameters and could vary from their anatomically based initial values. The optimization results were found to be in reasonable agreement with (scarce) experimental data from the literature (from a different type of bird) in terms of muscle fibre length (i.e. model “contractile element” length) over time (quantified by sonomicrometry) and muscle activation over time (quantified by EMG). The normalized fibre lengths visited during these trials maximally ranged from 0.5-1.2, with some muscles using a greater portion of this range than others.

Next, the authors randomly chose 5000 static poses for a reduced (6 DOF) version of the lower limb model, spanning the full range of motion of this 6 DOF model. For each of these poses they solved various versions of the muscle load sharing problem to establish the maximal range of fibre lengths that could conceivably be visited. The range of fibre lengths visited in all poses was compared to the common presumption expressed in the literature that normalized muscle fibre length ranges from 0.5 to 1.5 over the full range of motion of a joint. It was found that this presumption does not hold for many muscles in their model. Lastly, the authors evaluated to what extent changes in joint angles are linearly related to changes in muscle fibre length, i.e. to what extent non-constant moment arm, tendon and pennation angle influence this relationship. From their static simulation results, they computed the least squares optimal, linear transformation between muscle fibre length and joint angle, and report the differences between the linear model and the simulation results for each muscle. The linearity of this relation is left to the reader to evaluate. Their results may have implications for the estimation of optimal muscle fibre length in extinct/extant species.

Major comments:

It seems that the authors very carefully conducted each analysis step. However, there were very many steps. It is not clear to me to what extent arbitrary choices throughout the analysis may have influenced the results, especially considering the limited amount of experimental data that was available. Can you make a case for why the results of this study are nonetheless reproducible? For example: Missing sections of kinematics sequences; L335-350. The description of three levels of hierarchy in filling in missing data suggest that all three levels were used/needed. Was it evaluated to what extent the decisions made during this process influenced the results? E.g. taking different stride portions to fill in these data? In addition, such an analysis would give some indication of the robustness of the results.

We completely sympathize with the Reviewer’s concern and it was (and is) a key concern of ours. The bare fact is that we simply do not have additional data to rigorously or quantitatively evaluate these at the present time; for example, in the approach of filling in gaps of data to create a complete stride cycle, we were frequently left with little choice as to where we could source the extra data. Whilst we tried to be as objective and rigorous as possible (drawing upon our collective 35+ years of work with animal experimental data), we nonetheless acknowledge that our choices and approaches throughout could influence the ultimate results. Despite this, we are encouraged that the kinematic and kinetic results, and subsequently the results for muscle behaviour, are on the whole quite consistent with previously published data for other bird species, suggesting that at the very least our fundamental findings and conclusions are not wrong. In response to a comment from the other Reviewer, we have created a schematic overview of the many steps in our study as a new Fig. 1, and hopefully this figure can also help the reader gain a (qualitative) sense of how choices made at one step of the study may have downstream consequences.

Reserve actuators 1) I agree with your interpretation that the reserve actuators may represent moments generated by ligaments or joint capsula that were not explicitly modelled (L1290-1300). If these indeed represent passive structures, and given that we are considering near periodic motion (I think, but see minor point ahead), the net work done by these reserve actuators during one complete stride should at best be zero or if anything slightly negative. Can you check in your results if the work done by these reserve actuators during a complete stride is indeed close to zero/negative, ie the reserve actuators represent some (unmodelled) conservative / dissipative force?

We agree theoretically, that if these reserves do indeed represent unmodelled passive structures, then the integral of the reserve power–time curve should be ~zero for a single, complete stride. However, there are at least two problems with practically testing this hypothesis using the current results.

Firstly, the numerical approach used to solve the dynamic optimization problem renders aspects such as work and power difficult to accurately quantify, unless special care is taken. In particular, we used a relatively coarse discretization of the simulation duration (100 mesh intervals/101 nodes for the entire trial, and hence less than this for a single, whole stride). Moreover, reserve torques were applied as piecewise constant functions across each mesh interval, in contrast to the muscle forces that are allowed to vary (from collocation point to collocation point). The collective effect of these factors means that much higher mesh resolution is required to accurately estimate power and (especially) work, which is something we were not focusing on at the outset of our study. Moreover, the very small size of the bird (potential energy of the centre of mass when standing, *mgh* ≈ 0.786 J) means that it is even more difficult to recognize if net work is ‘approximately zero’ or not.

A second problem is that the reserve actuators in the current optimization formulation did not have activation-deactivation dynamics (they were applied instantaneously at each point in time), and moreover their contribution to joint moment balance at any given point in time is only related to joint moment balance at other points in time through the collective term in the objective function (summing across all time steps). That is, for all intents and purposes they are instantaneous entities that act independent of previous or future moments produced by themselves or muscles. Consequently, there is no mechanistic reason to expect that these reserve actuators should in fact perform zero net work across an integral number of strides (regardless of the level of mesh refinement), and hence no way to currently distinguish between whether the reserves represent passive structures or model deficiency (e.g., weak muscles), or both.

To properly explore the question of passive structures and their contribution to joint moment balance in the future, we believe this would require a mechanistic model of passive force generation to be implemented in the optimization routine, and the resulting problem solved at a much higher mesh resolution than used here. The results of the present study at the very least pinpoint those degrees of freedom where future research efforts should be targeted in this regard. We have distilled the above points into a brief note into the Discussion section (p. 53, line 1393-1400).

Reserve actuators 2) L945-950. Apparently producing the required joint moments using muscles alone, even for DOF’s that were well-spanned by the muscles, resulted in unrealistic results. This might be viewed as an important indication that something is missing in the model, e.g. maximum forces may have been too low, or something else might be missing. What are your thoughts on this?

The ‘unrealistic results’ we were referring to was in primary reference to the ankle extensor and flexor muscles. Muscles from both groups (e.g., EDL and TC [flexors], GL and FP4 [extensors]) span both the knee and ankle, and consequently they needed to balance at least six degrees of freedom. For some of these DOFs (especially ab–adduction), the muscles’ moment arms were really small; thus, to balance these non-flexion–extension DOFs (with less help from reserves) required higher muscle forces, which in turn has a knock-on effect for moment balance about other DOFs in these joints. Hence, at the present time we think that the key cause for our observation is the multi-articular nature of these muscles, which in life are used principally to control just flexion–extension of the knee and ankle.

Fmax estimation. L1230-1240, in relation to figure 4. In figure 4, the activation seems to ‘clip’ or nearly clip for some muscles during the stance phase, which might be the reason why reserve actuators have to be invoked. Apart from the latter consequence, this also suggests that the running trial represented an activity that is close to the maximum effort capability of the animal. Considering the animal was moving in more or less steady state at moderate speed, this seems highly unlikely, and suggests that for instance your maximum force values might be underestimated, or some other (dynamical) muscle properties are misestimated. Do you agree with this? If so, where in the model do you suspect modifications are required? (Note that your maximal force values depend on L0. The simulation based estimated values for L0 were higher than the anatomical based L0, which were used to compute the estimates for maximum force. I believe this means basing maximum forces on simulation based L0 values would if anything decrease the Fmax values.)

In the interest of not introducing one further assumption into the process (which was already laden with assumptions and simplifications), we decided to keep our Fmax values unaltered, which at the very least for the current study increases its biofidelity. But we agree, faster speeds or more strenuous activities would probably necessitate a greater Fmax for our muscles. Indeed, as noted in the cited paragraph (and elsewhere in the manuscript), previous studies of human running have often found that they need to use a higher Fmax (corresponding to a higher fibre stress) to achieve fast running speeds. This issue highlights a recurring theme in musculoskeletal modelling: there is a discord between strict anatomy-based estimates as fed into a Hill model and what real animals can actually do in maximal performance behaviours. We are currently in the process of exploring this topic in predictive simulations of maximal speed running and vertical height jumping with tinamou, but are not yet in a position to be able to comment any further on this point.

Minor comments:

To facilitate comparison with other literature/species, consider (also) reporting non-dimensionalized values throughout the manuscript (e.g. non-dimensionalized running/walking velocity, ground reaction force, etc.)

Done.

Do you have any indication to what extent the surgery (6 days recovery time) influenced the walking pattern of the tinamou, e.g. comparison of video data before/after surgery?

Only in a qualitative sense; general observations of the birds indicated no apparent lameness. We have now stated this on p. 10, line 261-262. The 6 days recovery time is pretty standard for bird locomotion studies (and some research groups, e.g., at Brown University, give even less time before commencing experiments).

Fig2. For both L* and Lt the scaling values are >1. Is this an indication that the muscle’s origin-insertion lengths in the model (for a similar anatomical pose) were on average larger than the origin insertion lengths during the anatomical measurements? In other words, could this result have been different if the muscles were a bit ‘stretched’ during the anatomical measurements?

It is possible, but there are a number of factors that could produce this discord, so we refrain from making any interpretation at the current time. Measuring total MTU length in dissections is notoriously difficult, especially for muscles that aren’t nice and fusiform in shape (and many avian hip muscles are fan- or ‘ball’-shaped), and probably more so for small animals. There is also the issue of the Hill model itself, representing a whole muscle as essentially a single fibre; indeed, as noted in reference to this figure, we suspect this to be the case for at least the ITC, which in reality is football-shaped and highly pennate. We also don’t know the resting sarcomere lengths of the muscles, which affects how accurate our assumption of optimal fibre lengths being equal to measured fascicle lengths really is. The tuning factor could also reflect an egregiously wrong value for assigned tendon stiffness. Collectively, these issues raise interesting and important questions about how well musculoskeletal models can honour real anatomical data, and we hope that our study can in the future help provide insight to improving the biofidelity of these models. We have briefly raised this issue in the Discussion section (p. 49, line 1315-1323).

L537; Vmax and Fmax were the same for all muscles, but there may exist some between-muscle variance in dynamic response, for instance due to differences in fibre type distributions. Can you make a case against the alternative hypothesis that the differences in (optimized) morphological properties between muscles do not reflect real morphological differences but are due unmodelled differences in the dynamical properties of the muscles?

That is an excellent point: in our study we only optimized two structural aspects of our model, but could alternatively (or even simultaneously) have been optimizing aspects related to fibre histochemistry (such as maximum contraction velocity, i.e., Vmax); we have included the issue of muscle-specific variation in Vmax in our above discussion of tuning factor results (p. 49, line 1315-1323). We had to start somewhere, and in lieu of a more comprehensive understanding of how dynamic properties vary within or across bird species (in terms of hindlimb musculature), we focused here on the structural aspects that we could readily measure. Nonetheless, with our model and data being made available on publication, this makes it possible for others to explore different approaches to optimization in the future.

L1124. For L*<0.5 the force producing capacity is ~0, and thus the force, and in particular tendon length (and thus L*) is dependent on the (arbitrary) numerical implementation of 0 muscle fibre force. Considering this, are the results for these 6 muscles meaningful? If not, consider removing them.

We agree that the result of having a super-short fibre length producing negligible muscle force is, biologically speaking, not particularly meaningful and almost certainly would not be used during *in vivo* behaviours. (Note that in the muscle model used [De Groote et al., 2016], the construction of the active force-length curve as a sum of three Gaussian curves means that active fibre force is always nonzero, even if it is really small.) We nevertheless included these results for the sake of completeness because this particular figure is more about what the model was capable of achieving in its totality, with respect to the aim of testing how well fibre lengths are reflective of joint range of motion. We have now clarified this point (p. 45, line 1183-1186).

L564: total body mass increased by 2.1 % to resolve dynamic inconsistency; this is surprising as I expect both your scale (on the basis of which animal mass (table 1) was determined) and your force plate to have an accuracy that is better than this 2.1 % BW. Could you explain?

Actually, we were surprised by how *little* mass had to change here, for a number of reasons. The mass of the birds will have fluctuated significantly over the course of the experiments (as does that in most experimental animals), and so mass on the day of data recording could have differed from the mass at the time of post-mortem measurement. Moreover, model mass was estimated using a digital method based on CT-derived volumes for a second individual and segment-specific densities derived from a third individual. The forceplates we used were old and also prone to a small amount of drift over the course of experiments (which we nevertheless tried to correct for in the post-processing stage). Lastly, there could easily have been stride-to-stride variation (and even step-to-step variation; Andrada et al., 2015, Kambic et al., 2015, which we already cite) in limb kinematics and ground reaction force production. Collectively, these factors can contribute (in animal experiments at least) to much greater dynamic discrepancies than those encountered here; in a similar project we are currently working on with crocodiles, preliminary results have shown a dynamic consistency discrepancy of >10% for some trials.

Table 2. I suspect that for the given methodology the number of significant digits provided for the variables in this table may not represent the accuracy / degree of certainty by which these values were measured / estimated (e.g. anatomically measured pennation angle accuracy of 0.01 deg, anatomically measured fibre length accuracy of 0.01 mm, or Fmax estimation accuracy of 1 mN…). If this were to be the case, please correct the number of significant digits accordingly.

That is correct, although for fibre lengths and pennation angles the reported values are the means of multiple measurements for each muscle (hence the extra digits). We have now rounded down all length and angle data in this table to one decimal place.

L611; 1) Here the modifications to GRM are not reported, which you may argue represent some dynamic inconsistency, or at least a modification to COP trajectories. Please also report the magnitude of these modifications. 2) “The greatest modification …” is not precise, as that refers to the greatest modification in terms of the RSME. Please correct so the reader understands that the greatest modification at any time node could have been larger than 10 % BW, or report this greatest modification (as the RMSE values are already reported in the table).

Point 1: As is true of any simulation using contact spheres (the Hunt-Crossley-Hertz model in Simbody; Sherman et al., 2011), our resulting gaits from the tracking simulations have GRMs *in all three directions*, whereas in reality feet can only exert moments on the ground about the vertical direction. The fact that we don’t have an apples-to-apples comparison here was the reason that we refrained from trying to track the GRMs in the simulations (in addition to the GRFs), and moreover is why we refrained from reporting modifications to the GRM in Table 3. Although our simulations are guaranteed to be dynamically consistent (since this is a ‘hard constraint’ in the tracking simulations), we do not believe that making a post-hoc comparison of GRMs is actually meaningful.

Point 2: Yes, we have now reported in the text the greatest instantaneous deviation in GRF for all three directions (p. 25, line 629-631).

Please provide a figure showing the (non)modified GRF’s as function of gait cycle. This will help the reader get a better appreciation of the data and facilitates comparison to (future) literature.

We have now reported the original (experimental) GRFs for the walking trial, along with the GRFs resulting from the tracking simulation, in a new Fig. S2. This not only provides the experimental data for future literature comparison, but it also illustrates the efficacy with which the tracking simulation followed the data (as per the previous comment). Since the GRF was not measured experimentally in the running trial, and our prediction of it (for Fx and Fy only) was based on previously published data as per Bishop et al. (2018), we have decided to not report the input curve.

L746. You mention the optimal control problem was subject to state continuity across trials. What does this mean? I initially thought this means the state variables were periodic, but looking at Figs 4 and 5 this does not appear to be case. Also, I am not sure whether the kinematics/kinetics of the skeletal part of the model are (enforced to be) periodic (as suggested by the kinematics cartoons)? Please clarify.

By ‘state continuity’, we simply were referring to continuity in terms of the direct collocation numerical approach: states at the end of one mesh interval (derived via Lagrange quadrature) match the states at the beginning of the next mesh interval, i.e., defect constraints (Betts, 2010) are zero. We did not impose any cyclicity constraint at all on the simulations, so any apparent near-cyclicity reflects the genuine near-cyclicity of the *in vivo* behaviours themselves. This point has now been clarified (p. 30, line 781-782).

L754-780. I appreciate the elaborate justification of the weight factors used. To facilitate comparison between them consider rescaling the weight factors in this section to similar dimensionless units, e.g. in comparing activation (term 1, ranging for 0-1) to reserve actuators (term 2) it might help to scale the weight on the reserve actuator force/activation to maximal reserve actuator force, or maximal joint moment or similar.

We think we understand what the Reviewer is saying here. Firstly, at least in our framework, scaling the weighting factors to dimensionless units is not advisable: different absolute values of the weighting factors (but which are nevertheless in the same proportion to one another across the terms) can produce different results; this is (probably) due to the way in which the objective function and constraint deficits are scaled within the nonlinear program solver. Regarding the second point, our term in the objective function pertaining to reserve recruitment also refers to activations that can range from 0 to 1; these activations are scaled within the optimization code (see code in supplementary material) using DOF-specific values that reflect the maximal external joint moment in the behaviours. So yes, ultimately, our reserve term is scaled in a fashion along the lines suggested by the Reviewer. We have now clarified this second point (p. 31, line 794-795, 797-800).

L800-820; please state explicitly that this results in a 6 DOF joint space from which the 5000 random positions were drawn.

Done.

Fig 6. Please indicate how these distributions are normalized; there is no scale on the y-axis.

There is not meant to be a scale on the y-axis here as these are simple histograms. The height of each figure is simply scaled by the magnitude (number of counts) of the most frequent bin in the histogram, and each muscle’s histogram is scaled to the same height for ease of visualization. Thus, muscles with much narrower histogram distributions (e.g., ILPOa) will have a higher-valued y-axis (greater number of counts in each bin). This has now been clarified in the figure caption.

L1140-1160. Differences between delta Lmt and delta Lm can be due to either pennation angle or tendon elongation. Please also mention the latter. Which of these two has the biggest contribution to the difference between delta Lmt and delta Lm? (perhaps this question is implicitly addressed in fig 7B., if so please make this clear)

Yes, we have now not only mentioned the effect of tendon elongation (p. 46, line 1215-1216), but also included an additional panel in the figure (new Figure 9), which compares the difference between MTU length change and fibre length change to the ratio of tendon slack length to fibre length (a measure of relative tendon length). This clearly demonstrates the effect of longer tendons, in addition to pennation angle.

Fig 7A 1) In the current figure it is not clear for which muscle’s there is a large discrepancy between delta_Lmt and delta_Lm. Consider plotting (in light of the question to what extent delta_Lm can be inferred from joint range of motion) the difference between delta_Lmt and delta_Lm, normalized by L0, for each muscle. Alternatively, consider adding labels to the points most saliently away from parity. 2) I would not expect the solutions for minimizing (sim1) and maximizing (sim2) activation to be similar (unless the resulting activations were similar), because higher activation implies higher force which results in more tendon elongation; I would thus expect the results for sim2 to be further away (more down) from parity. Similar argument goes for sim3 and sim4. Consider showing all results, or the most extreme results here.

Point 1 – We have now added labels to the data points to indicate which muscles deviate furthest from the line of parity (new Fig. 9). Our current plot design already illustrates the difference between change in MTU length versus change in fibre length: the further a data point plots below the line of parity, the greater the difference. We have also color-coded the data points based on the order in which muscles are listed in Table 2, which is arranged approximately proximal-to-distal. Thus, collectively the three panels in Fig. 9 show how more pennate or longer tendoned muscles experience greater discrepancy, and this tends to occur more towards the distal limb.

Point 2 – As noted in the caption the results actually produce a very similar plot for each simulation:

We therefore remain skeptical of the value of showing all the results, for all four simulations, which would add unnecessary clutter and potentially confuse the reader.

Fig 7B. I assume the grey line shows the linear fit. Consider plotting instead the theoretical relationship with pennation angle, for a non-compliant tendon, in which case the scatter would be due to variations in tendon length / force.

Conceptually this is an excellent idea. However, deriving the theoretical relationship would be exceedingly difficult – if not impossible – because the data points ultimately reflect the results stemming from 5,000 random (i.e., non-deterministic) poses, and the effects of other muscles (and their specific architectures and strengths) on how each muscle equilibrates for each pose. It is (unfortunately) not a matter of simply taking values for fibre length, slack length and pennation and deriving a nice equation relating change in MTU length to change in fibre length. For a single muscle system we could do that, but not one with 36 muscles.

L1156. The reference to differences with the line of parity in A is confusing here as these differences arise both from tendon compliance and pennation angle. If the point of Fig 7B is that higher pennation angles leads to greater decoupling between Lmt and Lm I would suggest making this statement in words rather than in a figure, as this observation follows directly from the formulation of the muscle model.

See comment above – we have now addressed this issue with a new panel in the figure.

Table 4. Please add means (sd) across the columns of this table; this may help the reader get a general sense of the data quickly.

Done.

L1213-1228. I appreciate the consideration for the future abilities of this new tuning approach. Please consider adding a cautionary note for the uninitiated reader, something like: ‘this new tuning also affords the possibility of patching errors made elsewhere in the procedure, and unintentionally reaching spurious conclusions’, or something along similar lines.

Done (p. 49, line 1298-1300).

L1364. “ … further paints a picture of structural conservatism across evolutionary timescales”. This statement is not strictly supported by data from the current study, please provide appropriate references.

What we were meaning with this (admittedly hand-wavy) statement was that in a comparative context muscles seem to function in a broadly similar fashion for all vertebrates, *suggesting* functional conservatism in an evolutionary context. We have better clarified the passage in question (p. 55, line 1458).

Fig1. (legend) Please indicate which panel the scale bar serves.

Done.

Language:

Please re-check the paper thoroughly for English. At various points, the text could be more concise. For example:

All the below points have been attended to as appropriate.

L99; “muscles … coordinate” many would argue it is the brain or central nervous system which does the coordinating, consider reformulating.

L116; “such research”, it is not clear (at this point what) “such” is referring to, consider: ‘such experimental research’

L122; perspectiveS

L127; “muscles have been able to be” convoluted language, consider reformulating, eg ‘muscles have been classified according to … ‘

L135; remove “constituent”

L173. “In particular, this would test”. Unfortunate formulation, consider rewording, eg ‘These results demonstrate / using these results, we can test whether …’

L212; “recovered” I guess it didn’t exist before, consider rewording, eg ‘produced’?

L602-603; correct sentence ending in “zero residuals”.

L590; spell out GRF/GRM at first use (which may have been before this one).

L653; “external joint moments”. Imo the use of the word external could be confusing here, because external moments might be thought to refer to some moment acting from the environment onto a system, whereas joint moments per definition are moments acting from one segment onto another segment within the same skeletal system. Also, the contrast with internal might not be so clear. Consider rewording, eg muscle generated moments (if you want contrast with reserve actuators).

L787. Remove “then”.

L798. Convoluted language, consider reformulating, eg “the greatest range of physiologically possible muscle lengths …”

L809. “control”, consider: ‘actuate’.

L989. “other” 🡪 otherS

L1012. Modellling 🡪 modelling

L1031. add ‘are’.

L1175. Consider: ‘fitting was done on the results from simulations 1 and 2 together, and on …’

L1349. “… have been able to be …” consider: ‘were’
