# Supplementary material for: Computational modelling of muscle fibre operating ranges in the hindlimb of a small ground bird (Eudromia elegans), with implications for modelling locomotion in extinct species
Source: PLoS Comput Biol. 2021 Apr 1;17(4):e1008843. doi: 10.1371/journal.pcbi.1008843 (PMC8016346; doi:10.1371/journal.pcbi.1008843)
Supplement: Attachment — Submitted filename: review_Bishop_etal_PlosCompBio2020_R1.docx [file pcbi.1008843.s011.docx]

Apart from the below point, the authors have adequately and in full replied to all questions and points raised. Congratulations with this good piece of work.

Initial reviewer comment:

L611; 1) Here the modifications to GRM are not reported, which you may argue represent some dynamic inconsistency, or at least a modification to COP trajectories. Please also report the magnitude of these modifications.

Author reply:

Point 1: As is true of any simulation using contact spheres (the Hunt-Crossley-Hertz model in

Simbody; Sherman et al., 2011), our resulting gaits from the tracking simulations have GRMs *in all three directions*, whereas in reality feet can only exert moments on the ground about the vertical direction. The fact that we don’t have an apples-to-apples comparison here was the reason that we refrained from trying to track the GRMs in the simulations (in addition to the GRFs), and moreover is why we refrained from reporting modifications to the GRM in Table 3. Although our simulations are guaranteed to be dynamically consistent (since this is a ‘hard constraint’ in the tracking simulations), we do not believe that making a post-hoc comparison of GRMs is actually meaningful.

Reviewer reply:

I disagree with this reasoning. The GRM represents a moment acting from the world on the model, which is needed to satisfy the rotational equation of motion for the model as a whole. The fact that, as you mentioned, the components of this GRM acting about the axes in the horizontal plane cannot exist in reality, means that they represent some 'residual' term in the model. Alternatively, a ground contact model could have been formulated in which the components of the GRM about the horizontal plane axes would have been zero. Compared to the current optimization results, this would have required changes in kinematics and ground reaction forces in order to maintain dynamic consistency. Given that the optimization results in terms of kinematics and ground reaction forces are not independent of the GRM’s, it merits the reader to know how large these optimized GRM’s are compared to measured GRM values.

I realize that the effect of constraining the GRM’s on the optimized kinematics and ground reaction forces are hard to predict, at best. Nevertheless, it might be helpful to point the reader to this interdependency between GRM’s, ground reaction forces and kinematics, as this may be a difficult point to grasp.

As an aside, I was unable to find in the cited reference where the moments arise in the contact model; it seemed to me that only contact forces are discussed therein. If that is correct, please provide the correct reference in which both the treatment of contact forces and moments are discussed.
