# Supplementary material for: Computational modelling of muscle fibre operating ranges in the hindlimb of a small ground bird (Eudromia elegans), with implications for modelling locomotion in extinct species
Source: PLoS Comput Biol. 2021 Apr 1;17(4):e1008843. doi: 10.1371/journal.pcbi.1008843 (PMC8016346; doi:10.1371/journal.pcbi.1008843)
Supplement: Attachment — Submitted filename: Bishop_Responses_to_Reviewer_Comments.doc [file pcbi.1008843.s013.doc]

**Responses to Reviewers’ Comments**

We thank the reviewers and editor greatly for their additional helpful comments. The original comments are listed below, and our response to each of these is given immediately after in blue font. Sections of the text where changes have been made in response to these comments are highlighted in yellow.

**Editor**

I agree with the reviewer that it may make sense to remark on the GRM modifications or comparison, and do not see why the authors say that such comparison "is not meaningful". Also, isn't it is not technically true that feet can only produce moments against the ground in the vertical direction: it's just that the moment about the vertical axis is the only one that can be very different from zero.

**Reviewer 1**

Apart from the below point, the authors have adequately and in full replied to all questions and points raised. Congratulations with this good piece of work.

Initial reviewer comment:

L611; 1) Here the modifications to GRM are not reported, which you may argue represent some dynamic inconsistency, or at least a modification to COP trajectories. Please also report the magnitude of these modifications.

Author reply:

Point 1: As is true of any simulation using contact spheres (the Hunt-Crossley-Hertz model in

Simbody; Sherman et al., 2011), our resulting gaits from the tracking simulations have GRMs in all three directions, whereas in reality feet can only exert moments on the ground about the vertical direction. The fact that we don’t have an apples-to-apples comparison here was the reason that we refrained from trying to track the GRMs in the simulations (in addition to the GRFs), and moreover is why we refrained from reporting modifications to the GRM in Table 3. Although our simulations are guaranteed to be dynamically consistent (since this is a ‘hard constraint’ in the tracking simulations), we do not believe that making a post-hoc comparison of GRMs is actually meaningful.

Reviewer reply:

I disagree with this reasoning. The GRM represents a moment acting from the world on the model, which is needed to satisfy the rotational equation of motion for the model as a whole. The fact that, as you mentioned, the components of this GRM acting about the axes in the horizontal plane cannot exist in reality, means that they represent some 'residual' term in the model. Alternatively, a ground contact model could have been formulated in which the components of the GRM about the horizontal plane axes would have been zero. Compared to the current optimization results, this would have required changes in kinematics and ground reaction forces in order to maintain dynamic consistency. Given that the optimization results in terms of kinematics and ground reaction forces are not independent of the GRM’s, it merits the reader to know how large these optimized GRM’s are compared to measured GRM values.

I realize that the effect of constraining the GRM’s on the optimized kinematics and ground reaction forces are hard to predict, at best. Nevertheless, it might be helpful to point the reader to this interdependency between GRM’s, ground reaction forces and kinematics, as this may be a difficult point to grasp.

As an aside, I was unable to find in the cited reference where the moments arise in the contact model; it seemed to me that only contact forces are discussed therein. If that is correct, please provide the correct reference in which both the treatment of contact forces and moments are discussed.

We thank the reviewer for this added discussion. Upon further investigation of the contact model, and reflection of our original response, we wish to provide the following clarification. In the contact model used here (the ‘default’ for OpenSim, albeit made algorithmically differentiable as per Falisse et al., 2019) only forces are actually computed (hence moments are not explicitly mentioned by the Sherman paper, as the reviewer points out), but when it comes to expressing the contact reaction as an output, moments are provided as well, expressed as if the point of application was at the foot segment’s origin. That is, OpenSim provides a 6-dimensional output equipollent with a 3-dimensional (force-only) output. This is what we meant when we said that our result contains GRMs in all three dimensions. Of course, as the reviewer noted in their first review, this is likely attendant with a different COP trajectory compared to that which was experimentally recorded, since in the simulations the COP is prescribed by the kinematics of the contact sphere(s), as determined by pes kinematics.

We agree that highlighting the interdependency of GRFs, GRMs and COP in our simulation approach would help the reader better understand the issue (have done this now on page 25, lines 634–641), and furthermore have now reported the RMS error for the vertical moment (simulation versus ‘free vertical torque’ as derived from the forceplates) in Table 3 [0.011 Nm for left pes, 0.03 Nm for right pes]. In future studies, especially where *n* > 1 and more detailed attention is given to foot–ground interactions, a more sophisticated contact modelling approach could be employed.

In regards to the related query raised by the editor, we concur that the feet can technically produce non-zero moments about the horizontal axes (small-scale friction/sticking effects, etc.); however, our forceplate setup and method of data processing (see MATLAB code provided in the SI) does not allow these horizontal moments to be parsed from horizontal moments induced by applied forces. Furthermore, even if we were able to obtain measurements for the actual horizontal moments applied by the feet, the quality of our data recording (as outlined in our prior revisions) and the small size of the bird means that it would be nigh impossible to distinguish real, non-zero signal from noise.

**Reviewer 2**

Thank you to the reviewers for the responses. I feel that the changes have improved the paper’s readability. As said before, the topic is very interesting. By the way, I was not able to see the yellow highlights in the manuscript.

Major comment:

“C1 [other reviewer]: The cut-off frequency when filtering the kinematic and kinetic data is different. According to [1], inconsistencies can arise when the cut-off frequency for kinematic and kinetic data is not the same. Please elaborate on the choice of cut—off frequency.

A1 [our response to this comment]: Our kinematic (XROMM) and kinetic (forceplate) data were obtained via very different means, rather than straight recording via a semi-automated motion capture protocol as is the norm in human studies. This consequently meant that the data had varying levels of ‘quality’ and noise. For example, the forceplates we used were quite old and prone to drift and noise, and so we erred on the side of caution and used a more aggressive filtering (lower cutoff frequency) for those data (clarifying statement now included on p. 15, line 393-397). We acknowledge that in a more ideal situation – and certainly one where we would be collecting data for many trials in a comparative analysis – a more consistent cutoff frequency would be preferred. Nonetheless, the cutoff frequencies that we ultimately used are similar to those used in previous studies of bird locomotion (e.g., Bishop et al., 2018), and ultimately our resulting kinematic and kinetic profiles are highly comparable to previously published data, so we have little concern with this aspect of the study.”

I think I mostly agree with the author’s response. However, at the same time I am not completely sure that we understand each other completely. Therefore, I have elaborated a bit more on this below:

*“rather than straight recording via a semi-automated motion capture protocol as is norm in human studies”* From this, I thought that the kinetics and kinematics were recorded in a separate trial. However, from the manuscript I understand that the kinematics and kinetics were measured simultaneously for the same stride. So I do not understand this part of the comment. Were the different recordings not synchronized? I think that that is the only difference between this approach and commonly used human motion capture that would affect the filtering issue.

Yes, *some* of the kinematic data were collected synchronously with the forceplate data for our focal trials, but as outlined in the text we had to source data from other trials to ‘fill in’ missing parts to recreate a complete kinematic sequence. (And, as noted in the text, the additional trials had to be synchronized manually to the focal trial, by virtue of foot touchdown and liftoff.) This is what we meant in referring to human motion capture studies: because of the scale and nature of the setup, a complete set of kinematic and kinetic data can be easily collected for entire stride cycles in humans, something we were unfortunately unable to do with our birds and in the small capture volume of our biplanar fluoroscopy setup.

Furthermore, the issue with having a different cut-off frequency for motions and forces, is that in this case you will filter out force peaks that happen at e.g. 18 Hz (the cut-off frequency is 15 Hz), while the corresponding 18 Hz motion is still there (because the cut-off frequency is 20 Hz). This will cause inconsistencies in the inverse dynamics results, because the motions that go into the multibody equation might not correspond to forces that go into the same equation. Furthermore, when data is not synchronized or a different time delay is introduced due to different cut-off frequencies, this would be even worse, because then the timing of motions is off. For example, in human gait, foot flat after heel strike would have to be done actively if the corresponding ground reaction force is not present, which could greatly affect joint moments. I suspect that this filtering inconsistency might also partly cause the high results in the residual reduction algorithm. However, I would say that this approach is fine for the optimal control problem, because there the experimental results are not directly inserted into the equation but tracked as best as possible.

We thank the reviewer for this added explanation, and have now made a note in the relevant part of the text highlighting how in future studies that have access to larger numbers of trials (and of higher quality), using a consistent cutoff-frequency would be desirable (page 15, lines 397–399).

Minor comments:

A couple of times, I was a bit confused because so many processing steps are required, and I got lost a bit about exactly what was used where.

- Residual reduction algorithm: why change the mass when this is actually measured exactly? Or was this only measured for the bird used for modelling and not for kinematics? Also considering the comment above, I am wondering a bit if you are correcting a measurement error by adapting the model, so that the residual reduction algorithm cancels out one error by introducing another error.

As outlined previously, the mass of the birds would have fluctuated on a daily basis and likely differed between the time of experiment and the time of post mortem weighing; also model mass was estimated using a digital method based on a second bird not used in the experiments. We therefore felt it entirely justifiable that mass be allowed to alter in the RRA (and, as outlined before, the resulting change of 2.1% was relatively small). We agree that allowing mass to be modified may have masked the correction of a different error in the system causing dynamic inconsistency, but this is ultimately unavoidable in an optimization problem with multiple things being modified at the same time (here, kinematics and mass properties); one aspect influences the other. With a greater sample size (i.e., *n* > 1) and a focus dedicated to exploring the outcome of different weighting approaches, how much one aspect of RRA influences the results of another aspect could be discerned.

- Did the optimal control use the model that was changed after residual reduction?

Yes; we have now clarified this in the relevant section (page 24, lines 599–600).

- Line 676-678: How did the optimal control produce a musculoskeletal model with altered mass properties?

Sorry, that was sloppy wording on our part; the bit about modified mass properties was referring to the previous section on RRA. We have now clarified the passage in question (page 27, line 693–696).

- Equation 4, line 796-802. You chose a high weight to ensure that reserve actuators were used as little as possible. Did you check if reserve actuators were used when activation was not yet maximal?

Yes; as in the main results presented in the manuscript, many muscles had submaximal activations (even in running) and reserve actuators were still being used when lower weightings were used. We essentially tried to push the model to its limit by forcing it to use as little reserve actuation as possible.

- Line 1000 – 1006: based on the reviewer comments that I had already read, I felt that here it would make sense to already add that these moments are caused by passive tissues, but then I noticed that this was addressed later on in the manuscript. However, since the manuscript is rather long, it might be helpful for future readers to connect these two paragraphs a bit more.

We initially abstained from mentioning passive tissues to keep results and interpretations separated, but agree that at least briefly mentioning them here will help the reader overall, as suggested (page 39, line 1022–1024).

- Line 1072- 1076: again, here I was wondering if there was an explanation, which I later found in the discussion. Again, it might be helpful to connect the paragraphs in the results and discussion.

As for previous comment (page 41, line 1094–1096).

- Typo line 1188 (waking)

Oops, thanks for catching that! Has been fixed.

- Section “Static simulations”, line 1170: I got lost a little bit here with all the different “l”s. I think readability could be improved when full names “e.g. optimal fibre length” are added to the variable names.

Yes, we have now interspersed the full wording (“optimal/normallized fibre length”) throughout the passage in question to better aid the reader in keeping track.

- 1376 – 1379: would you conclude after this study that a one DOF ankle and knee is appropriate for musculoskeletal models of birds?

Good question! We think it depends on what the study’s goals actually are. In our case where we were focusing on gross muscle function across the scale of the whole limb, yes, such a simplification would be appropriate. But we can easily imagine cases where a more sophisticated approach would be important, such as a study focusing on a specific distal limb muscle, or focusing explicitly on the function of the passive tissues themselves, or examining more three-dimensional behaviours such as turning manoeuvres.
