# Supplementary material for: Computational modelling of muscle fibre operating ranges in the hindlimb of a small ground bird (Eudromia elegans), with implications for modelling locomotion in extinct species
Source: PLoS Comput Biol. 2021 Apr 1;17(4):e1008843. doi: 10.1371/journal.pcbi.1008843 (PMC8016346; doi:10.1371/journal.pcbi.1008843)
Supplement: Attachment — Submitted filename: Bishop_Responses_to_Reviewer_Comments.doc [file pcbi.1008843.s015.doc]

**Responses to Reviewers’ Comments**

We thank Reviewer 2 for their additional helpful comments, which have helped clarify an outstanding source of ambiguity in the text. The original comments are listed below, and our response is given immediately after in blue font. Sections of the text where changes have been made in response to these comments are highlighted in yellow.

**Editor**

The reviewer has asked for very minor edits/clarifications regarding the GRM calculation and comparisons. As the reviewer asks, it may be worth remarking how GRMs were handled more explicitly.

**Reviewer 1**

All my comments/questions have been answered. Congratulations on a great paper!

**Reviewer 2**

Please find below my final thoughts on the GRM issue. I’ve included the previous correspondence for reference first.

*Review round 1 – Initial reviewer comment: L611; 1)*

Here the modifications to GRM are not reported, which you may argue represent some dynamic inconsistency, or at least a modification to COP trajectories. Please also report the magnitude of these modifications.

*Revision 1 (manuscript v2) – Author reply:*

Point 1: As is true of any simulation using contact spheres (the Hunt-Crossley-Hertz model inSimbody; Sherman et al., 2011), our resulting gaits from the tracking simulations have GRMs in all three directions, whereas in reality feet can only exert moments on the ground about the vertical direction. The fact that we don’t have an apples-to-apples comparison here was the reason that we refrained from trying to track the GRMs in the simulations (in addition to the GRFs), and moreover is why we refrained from reporting modifications to the GRM in Table 3. Although our simulations are guaranteed to be dynamically consistent (since this is a ‘hard constraint’ in the tracking simulations), we do not believe that making a post-hoc comparison of GRMs is actually meaningful.

*Review round 2 – Reviewer reply:*

I disagree with this reasoning. The GRM represents a moment acting from the world on the model, which is needed to satisfy the rotational equation of motion for the model as a whole. The fact that, as you mentioned, the components of this GRM acting about the axes in the horizontal plane cannot exist in reality, means that they represent some 'residual' term in the model. Alternatively, a ground contact model could have been formulated in which the components of the GRM about the horizontal plane axes would have been zero. Compared to the current optimization results, this would have required changes in kinematics and ground reaction forces in order to maintain dynamic consistency. Given that the optimization results in terms of kinematics and ground reaction forces are not independent of the GRM’s, it merits the reader to know how large these optimized GRM’s are compared to measured GRM values. I realize that the effect of constraining the GRM’s on the optimized kinematics and ground reaction forces are hard to predict, at best. Nevertheless, it might be helpful to point the reader to this interdependency between GRM’s, ground reaction forces and kinematics, as this may be a difficult point to grasp. As an aside, I was unable to find in the cited reference where the moments arise in the contact model; it seemed to me that only contact forces are discussed therein. If that is correct, please provide the correct reference in which both the treatment of contact forces and moments are discussed.

*Revision 2 (manuscript v3) – Author reply:*

We thank the reviewer for this added discussion. Upon further investigation of the contact model, and reflection of our original response, we wish to provide the following clarification. In the contact model used here (the ‘default’ for OpenSim, albeit made algorithmically differentiable as per Falisse et al., 2019) only forces are actually computed (hence moments are not explicitly mentioned by the Sherman paper, as the reviewer points out), but when it comes to expressing the contact reaction as an output, moments are provided as well, expressed as if the point of application was at the foot segment’s origin. That is, OpenSim provides a 6-dimensional output equipollent with a 3-dimensional (force-only) output. This is what we meant when we said that our result contains GRMs in all three dimensions. Of course, as the reviewer noted in their first review, this is likely attendant with a different COP trajectory compared to that which was experimentally recorded, since in the simulations the COP is prescribed by the kinematics of the contact sphere(s), as determined by pes kinematics. We agree that highlighting the interdependency of GRFs, GRMs and COP in our simulation approach would help the reader better understand the issue (have done this now on page 25, lines 634–641), and furthermore have now reported the RMS error for the vertical moment (simulation versus ‘free vertical torque’ as derived from the force plates) in Table 3 [0.011 Nm for left pes, 0.03 Nm for right pes]. In future studies, especially where n> 1 and more detailed attention is given to foot–ground interactions, a more sophisticated contact modelling approach could be employed. In regards to the related query raised by the editor, we concur that the feet can technically produce non-zero moments about the horizontal axes (small-scale friction/sticking effects, etc.); however, our force plate setup and method of data processing (see MATLAB code provided in the SI) does not allow these horizontal moments to be parsed from horizontal moments induced by applied forces. Furthermore, even if we were able to obtain measurements for the actual horizontal moments applied by the feet, the quality of our data recording (as outlined in our prior revisions) and the small size of the bird means that it would be nigh impossible to distinguish real, non-zero signal from noise.

(main) Author text addition:

Deviation between the vertical GRM in the simulation and as measured experimentally (the ‘free vertical moment’) was on average less than 2% of the product of body weight and standing hip height, with a peak deviation of 7.8% of the product of body weight and standing hip height. It should be recognized that in the present simulation framework there exists an interdependency of GRFs, GRMs and kinematics, since the centre of pressure of the GRF (which in turn affects the computed GRMs as expressed in a local or global reference system) is dictated by the kinematics of the body segment to which the contact sphere is appended.

Review round 3 (current round) – Reviewer reply:

Thank you for this clarification. This is my current understanding of what the GRM represents in your modeling framework, please correct if that is wrong.

1) The GRF and its point of application is a function of the kinematics (through the foot contact model), and the kinematics of the other segments are constrained to satisfy linear momentum balance.

Yes, correct.

For given optimized kinematics, the moment of the GRF about the model CoM will in general not equal the rate of change of angular momentum about the model CoM, giving rise to a ‘residual moment’.

2) The GRM acting from the ground on the model is now made to equal the discrepancy in the angular momentum balance equation (taken about the model CoM), such that the residual moment becomes zero. (I assume this is what is meant with the phrase “the GRM’s were allowed to vary freely”?). All the joint torques etc. are made to accommodate this GRM acting on the foot.

This is not correct. The GRM that the pes (contact sphere) experiences is not directly constrained/related to the residual moments at the ground–pelvis joint. Rather, as outlined in the supplementary code, the GRM (and GRF) is calculated purely and directly from the contact sphere’s kinematics; moments and forces then propagate up the kinematic chain to the root-most body (pelvis in this instance): the GRM is purely an input, not an output. Therefore, in general, for an arbitrary configuration of model coordinates (generalized positions, velocities and accelerations) there *will* be non-zero ground–pelvis residuals, but one of our path constraints is that in the solution these residuals are zero (within the tolerance of the optimizer – here set to 10^–4^). That is, that the optimizer adjusts kinematics and such to ensure moment balance, rather than doing anything directly with the GRMs themselves.

It is now apparent that this confusion stems from our statement that the GRMs “were allowed to freely vary”. What we meant by this statement was that there was no term in the objective function encouraging the tracking of experimentally recorded GRMs (unlike the GRFs, which were encouraged to track experimental records). We did not mean that GRMs were allowed to vary in direct response to the need to satisfy moment/angular momentum balance. We have now re-worded the phrase in question to avoid this ambiguity for the reader (page 24, lines 611–613).

If the above is true, the comparison between modelled and measured GRM is indeed complicated by the placement of the point of application of the modelled GRF at the foot origin. A way to still make this comparison, might be to move the point of application of the optimized GRF from the foot origin to whichever best (based on force plate data or kinematics or some combination of both) represents the measured CoP location (by adding the appropriate r ⃗×F ⃗_ground term to the GRM). This new GRM vector can now be meaningfully compared to the measured GRM, in all 3 dimensions.

I disagree with the assertion that the model’s GRM and GRF (OpenSim’s 6-dimensional output) is equipollent with a 3-dimensional force only output applied at an appropriate CoP. I believe this is most likely not the case, because the GRM’s were allowed to vary freely to satisfy angular momentum balance for the model as a whole.

As above, this inference is not correct. GRMs derive strictly from just the kinematics of the contact elements.

The residual moment that needs to be corrected for, may not be perpendicular to the GRF, in which case it cannot be corrected for by moving the point of application of the GRF. An easy way to check this, would be to evaluate the inner product of GRF and GRM. If this inner product evaluates to zero, then the GRM and GRF are equipollent to a displaced GRF. I expect this inner product to evaluate to non-zero, however.

We have now taken the results of the tracking simulation and computed the inner (dot) products as recommended by the Reviewer, which is reported in the graph below:

As can be seen, the results for both feet are less than the optimizer tolerance (10^–4^) from zero, and so numerically/effectively are indeed zero, contrary to the expectation of the Reviewer and supporting our original statement.

I concur with both the related comment by the editor and the author’s response to it.

Specific comments

L590 ground-pelvis joint. Is that correct?

Yes. This is a common term, at least in our experience, used to refer to the 6 degree-of-freedom connection between the root-most body in the model (in our case, the pelvis) and the world space (ground).

L611 “(GRM’s were allowed to vary freely to achieve dynamic consistency).” Consider removing the brackets here, and elaborating a bit more. Dealing with dynamic inconsistencies in the data involves two equations: linear momentum balance and angular momentum balance. I think that the method of dealing with discrepancies in the latter equation merits some justification, either on general grounds and/or by examining the effects quantitatively.

In light of the above discussion, yes, we have elaborated on this point and have provided additional clarification in the text (page 24, lines 611–613).

L617 “(and GRM’s)” as I understand now the GRM’s actually do not arise completely from the contact model (via displacement of the GRF), but their values are in part assigned to satisfy angular momentum balance for the given kinematics. Is that correct?

Not correct – as explained above (and in our response in the previous revision), the GRMs arise purely from the kinematics of the contact elements. They are not ‘back-derived’ so as to satisfy outstanding rotational moment (= angular momentum) balance.

L621, perhaps state here that GRF’s follow directly from the kinematics of the foot, given the ground contact model?

Yes, this additional clarification has now been provided (page 25, lines 622–623).

L622-623: Again, it seems to me that this only concerns dynamic inconsistency in terms of linear momentum balance. Angular momentum balance is dealt with separately, by applying a GRM on the foot that ensures angular momentum balance is satisfied given the kinematics and GRF?

Not correct; see our explanation above.

L634-637: Why only discuss the vertical component here? See suggestion above for meaningful comparison between modelled and measured GRM in all 3 dimensions.

As explained in our previous submission, comparison of horizontal moments is not possible because we were unable to compute these from our experimental records. In light of our additional explanations, the above suggestion is no longer relevant.

L640-641: The reader might not realize here that the kinematics of all segments contribute to potential discrepancies in angular momentum balance (e.g. by affecting location of body COM, direction and magnitude of GRF, rate of change of angular momentum, etc. etc.). As noted above, any displacement of the CoP can only correct for the component of this discrepancy that is normal to the GRF vector. I am by no means suggesting to list all possibilities, but I would make it a general statement, or make clear that you give a specific example that only concerns that component of the discrepancy in the direction normal to the GRF.

Yes, we had already made some statement to this effect previously, and have now expanded on it further in this revision (pages 25–26, lines 642–647).

L649-651: See remarks above.

Our above responses have now dealt with the topic covered in this part of the text.
